# Conservative and surgical management of foot and ankle sporting injuries in the weekend warrior

**DOI:** 10.1186/1757-1146-4-S1-I1

**Published:** 2011-05-20

**Authors:** Harvinder S Bedi

**Affiliations:** 1 Orthopaedic Department, Alfred Hospital, Prahran, Victoria, Australia

## 

A clinical based review of the injuries that are regularly suffered by Joan and John Average is presented. Common presentations of conditions such as lateral ligament tears, talar osteochondral lesions, syndesmotic injuries and 5^th^ Metatarsal fractures are discussed along with the management and prognosis of these problems. Questions are answered including how does it occur; what should I do about it; what can somebody else do about it and what occurs in the future?

